# Modulation of Molecular Biomarker Expression in Response to Chemotherapy in Invasive Ductal Carcinoma

**DOI:** 10.1155/2018/7154708

**Published:** 2018-02-12

**Authors:** Mana Oloomi, Neda Moazzezy, Saeid Bouzari

**Affiliations:** Department of Molecular Biology, Pasteur Institute of Iran, Tehran, Iran

## Abstract

Breast cancer (BC) has varied morphological and biological features and is classified based on molecular and morphological examinations. Molecular classification of BC is based on biological gene-expression profiling. In this study, biomarker modulation was assessed during BC treatment in 30 previously untreated patients. Heterogeneity among patients was pathologically diagnosed and classified into luminal and basal-like immunohistochemical profiles based on estrogen, progesterone, and human epidermal growth factor receptor (ER/PR/HER2) status. Marker heterogeneity was compared with mRNA biomarker expression in patients with BC before and after therapy. Reverse transcription-polymerase chain reaction was performed for molecular characterization. Expression and modulation of biological markers, CK19, hMAM, CEA, MUC, Myc, Ki-67, HER2/neu, ErbB2, and ER, were assessed after treatment, where the expression of the biomarkers CK19, Ki-67, Myc, and CEA was noted to be significantly decreased. Marker expression modulation was determined according to different stages and pathological characteristics of patients; coexpression of three markers (CK19, Ki-67, and Myc) was specifically modulated after therapy. In the histopathologically classified basal-like group, two markers (CK19 and Ki-67) were downregulated and could be considered as diagnostic biomarkers. In conclusion, pathological characteristics and marker variation levels can be evaluated to decide a personalized treatment for patients.

## 1. Introduction

Breast cancer (BC) is the most common cancer in women worldwide and is reported to be the second most common cancer in Iranian women [1]. Its progression is a multistep process with defined prognosis—from ductal carcinoma in situ (DCIS) as the final preinvasive stage to invasive ductal carcinoma (IDC) [2].

Tumor size and grade, ER/PR/HER2 receptor expression, lymph node metastases, and vascular or perineural tumor invasion are pathological categories generally used for defining the prognosis of BC [3]. Numerous other parameters, such as the proliferating index and P53, CK, HER1, or carcinoembryonic antigen (CEA) molecular markers, are also used for evaluating the prognosis and predicting therapeutic outcomes. Furthermore, proteomic immunohistochemistry (IHC) classification distinguishes between luminal and basal-like carcinomas based on the molecular marker's expression on the tissue [3–5].

The most common histological category is DC, which is a malignant breast tumor, followed by lobular carcinoma, while medullary carcinoma is relatively rare [6]. Infiltrating DC has been classified based on the molecular subtypes designated as luminal (ER/PR), HER2 overexpressing, basal-like (CK5/6+, EGFR+), and normal breast-like; each group has a different clinical outcome [7]. Responsiveness to treatment (neoadjuvant therapy) of patients in the basal-like group has also been previously reported [7]. Multigene genomic classification has been suggested to complement traditional pathological methods [8].

Gene-expression profiling has altered previous perceptions about BC and could be considered as a new molecular diagnosis tool [9, 10]. Moreover, a defined molecular marker coexpression panel for each patient with BC based on pathological characteristics and modulation assessment of expression level after treatment can provide personalized treatment for patients [11]. In this study, specific biomarkers, CK19, hMAM, CEA, MUC, Myc protooncogene, Ki-67, HER2/neu, and ER, in the serum of patients with BC were assessed before and after therapy. Significantly expressed molecular markers were considered for the assessment of marker variation before and after treatment. Marker expression and coexpression were also considered in different stages and pathological features with regard to markers detected by immunohistochemistry (HER2, ER, and PR) as a routine procedure. Patients with different stages of BC were divided into three pathological categories for analyzing biomarker expression and response after therapy.

## 2. Materials and Methods

### 2.1. Sample Collection

In this case-control study, 30 untreated female patients with BC and 30 healthy female controls were evaluated at Milad Hospital, Tehran, Iran, from 2012 to 2014. The healthy controls had no clinical history of malignancy or breast disease and were 31 to 56 years old (median = 45 years), the same age range as the patients. We compared biomarker expression before and after treatment in patients with the same treatment protocol. Written informed consent was obtained from each participant. This study was approved by the National Ethical Committee of Pasteur Institute of Iran (ethical approval number: 4552). The clinical history (age, tumor node, metastasis, stage, histological findings, and survival time) was collected for each patient and parameters are summarized in Table 1. Different stages of tumor (I–IV) in patients with solid cancer were classified according to the standard criteria based on tumor, nodes, and metastases (TNM) data and staging system of American Joint Committee on Cancer (AJCC). The heterogeneity among our patients was defined in Table 1. Most of our patients were diagnosed with invasive ductal carcinoma (IDC) and classified into luminal and basal-like immunohistochemical profile, based on ER/PR/HER2 molecular status.

All the patients received locoregional and systemic treatment according to the hospital protocol. The treatments were based on the World Health Organization (WHO) guidelines. Blood samples were collected after primary diagnosis, before the initiation of adjuvant chemotherapy, before surgery (phase I/before treatment), and after treatment during the follow-up period (phase II/after treatment). Peripheral blood (10 mL) from healthy controls and patients was obtained and collected in buffered sodium citrate and was incubated at 4°C within 2 h.

All the patients received the same treatment, were followed up for 3 years, and were still disease-free. The function of the biomarkers for detection of BC and their sequences are presented in Table 2 [12–18].

### 2.2. RNA Extraction and Quantification

Whole blood samples (10 mL in EDTA) from the patients and healthy controls were selected. From whole blood, RNA was isolated using the AccuZol™ (Bioneer, Korea). According to manufacturers' instructions of the manual procedure for fresh blood samples, 250 *μ*L whole blood was used in each round of RNA isolation. The RNA was extracted and assessed using a spectrophotometer. RNA concentration was determined by measuring the absorbance at 260 nm (A_260_). Purity of isolated RNA was analyzed by the ratio at 260/280 nm. RNA purification was assessed by ethidium bromide (EtBr) staining after agarose gel electrophoresis. EtBr (1 *μ*g/mL) was added to the agarose gel for RNA visualization. Then, the purified RNA was used to perform reverse transcription-polymerase chain reaction (RT-PCR).

### 2.3. RT-PCR

AccuPower® RT/PCR PreMix (Bioneer) kit was used for total RNA amplification. All the components necessary for cDNA synthesis and amplification were added in a tube. The gene expression of markers in the collected samples was screened in the peripheral blood. In this study, the oligonucleotide sequences were used, which have previously been reported [12, 17–19]. The RNA extracted template and the reverse primer were mixed in a sterile tube, followed by mixture incubation at 70°C for 5 min. Then the mixture and the forward primer were transferred to premix tube and filled with distilled water. The cDNA synthesis was conducted at 42°C for 60 min and at 94°C for 5 min. PCR for all the genes was performed for 30 cycles and the conditions were as follows: 94°C (60 sec), 54°C (30 sec), and 72°C (60 sec). The PCR products were separated by electrophoresis using 2% agarose gel and visualized in a gel documentation system under UV transilluminator. Glyceraldehyde-3-phosphate dehydrogenase (GAPDH), a housekeeping gene, was used to normalize the expression of the molecular markers as a reference gene.

### 2.4. Statistical Analysis

Student's *t*-test and Pearson's chi-square (*χ*2) test were performed to evaluate the correlation of mRNA biomarkers in peripheral blood samples. A value of *P* < 0.05  was considered statistically significant. Data processing was performed using SPSS software, version 18.

## 3. Results

### 3.1. Distribution of Biomarker Expression

Biomarker expression in patients with invasive BC was assessed. The patients with standard and partial mastectomies received treatment prior to surgery in phase I. Marker expression in patients diagnosed with BC was assessed 6 months after treatment.

**Figure 1:**
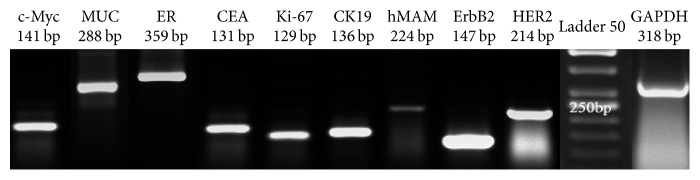
Representative image showing* Myc*,* MUC, ER, CEA, Ki-67, CK19, hMAM, ErbB2,* and* HER2* mRNA expression on 2.5% agarose gel after RT-PCR.

**Figure 2:**
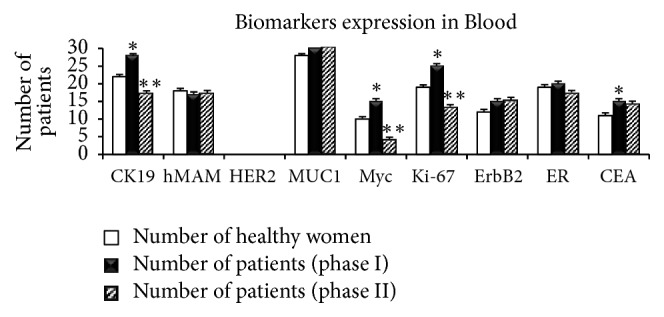
Expression of biomarkers analyzed in blood from healthy women and patients with BC in phases I (before treatment) and II (after treatment). ^*∗*^*P*  value ≤ 0.05 was considered significant between phase I and healthy women; ^*∗∗*^*P*  value ≤ 0.05 was considered significant between phase I and phase II.

If the tumor was node-positive, patients received systemic adjuvant therapy and anthracycline-containing chemotherapy. Distribution of biomarkers in different stages (I–III) after treatment (phase II) is shown in Table 3. Expression of* Myc*,* MUC*,* ER*,* CEA*,* Ki-67*,* CK19*,* hMAM*,* ErbB2*, and* HER2* mRNA markers was detected in patients with BC and compared with that in 30 healthy women using RT-PCR (Figure 1). A significant difference in* CK19 *(*P* = 0.012),* Myc *(*P* = 0.023),* Ki-67 *(*P* = 0.012), and* CEA *(*P* = 0.043) expression was found in phase I between the patients and healthy controls. The expression of* hMAM*,* MUC*,* ErbB2 (HER2*/*neu)*, and* ER* markers did not differ significantly between the two groups (*P* > 0.05, Figure 2). Variation in expression of* CK19*,* Myc*, and* Ki-67* markers between phases I and II was significant (*P* ≤ 0.001, Table 3(a)). Biomarker distribution before (phase I) and after (phase II) treatment is shown in Table 3(b) based on the different stages (I, II, and III). In phase I, the correlation between* hMAM*,* Myc*, and* ErbB2* mRNA expression and tumor stage was significant (*P* < 0.001), but no correlation was found between* MUC, ER, CEA, Ki-67, CK19,* and* HER2* and tumor stage. An association between* hMAM, Ki-67, ErbB2, ER*, and* CEA* mRNA expression and clinical stages (*P* = 0.034 and 0.013; *P* ≤ 0.001, 0.004, and 0.029, resp.) was noted in phase II (Table 3(b)).

### 3.2. Coexpression of Biomarkers and Its Association with Disease Stage

Based on Table 4(a), coexpression of positive biomarkers ((CK19, Ki-67, Myc, and CEA), (CK19, Ki-67 and Myc), and (CK19 and Ki-67)) was 6%, 6%, and 63% in healthy women, respectively. There were significant differences in the coexpression biomarkers ((CK19, Ki-67, Myc, and CEA) and (CK19, Ki-67, and Myc)) in healthy women versus those in patients in phase 1 (*P* < 0.05). There was also a significant difference in the coexpression of biomarkers (CK19 and Ki-67) in healthy women versus those in patients in phase II (*P* < 0.05). Coexpression of positive biomarkers ((CK19, Ki-67, Myc, and CEA), (CK19, KI67, and Myc), and (CK19 and Ki-67)) between patients in phases I and II was statistically different (*P* < 0.05).

**Figure 3:**
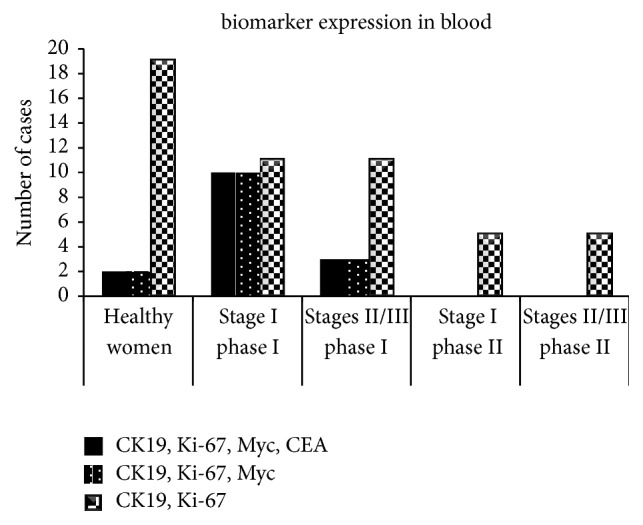
Coexpression of biomarkers analyzed in blood from healthy women and patients in phases I (before treatment) and II (after treatment) in different stages of BC.

Coexpression of biomarkers and its association with tumor stage were also considered (Table 4(b)). Coexpression of positive biomarkers (CK19, Ki-67, Myc, and CEA) was 77% in total in stage I (Table 4(b)). Two-marker variation was 50% in total in phase I. In phase II, coexpression of positive biomarkers was not observed in the three stages (Table 4(b)). In phase I, the correlation between four (CK19, Ki-67, Myc, and CEA), three (CK19, Ki-67, and Myc), two (CK19 and Ki-67) markers coexpression and stage was significantly different (*P* ≤ 0.001, 0.001, and 0.006, resp.), but no correlation was found between biomarker coexpression and stage in phase II. Variations in four and three markers were observed in 77% and 23% of the patients in stage I and stages II/III, respectively (Figure 3). It was shown that, in patients with stage I and stage II tumor, the expression of four and three significant markers was decreased. Therefore, assessment of positive significant biomarker coexpression (CK19, Ki-67, Myc, and CEA) in different stages should be considered.

### 3.3. Pathological Markers

In this study, we also evaluated clinicopathological features of patients with BC and molecular expression of CK19, Myc, and Ki-67 (Table 5(a)). Patients were categorized based on their pathological features as HER^+/−^ and ER/PR^+/−^. HER2^−^/ER^+^/PR^+^ (luminal A), HER2^+^/ER^+^/PR^+^ (luminal B), and HER2^−^/ER^−^/PR^−^ (basal-Like) biomarker expressions were considered and compared in phases I and II. Myc, CEA, and Ki-67 markers were mostly expressed in luminal A (66.7% and 60%).

The coexpression of CK19, Ki-67, Myc, and CEA markers was compared in phases I andII (Table 5(b)). Coexpression variation of CK19, Ki-67, Myc, and CEA markers in phase II was also observed in the three pathological groups. In phase I, no correlation was found between CK19, Ki-67, Myc, or CEA mRNA marker expression and pathological features (HER2^−/+^, ER^+^/PR^+^ or HER2^−^, ER^−^/PR^−^) (Table 5(a)). Four (CK19, Ki-67, Myc, and CEA) and three (CK19, Ki-67, and Myc) markers' variation was not observed in phase II. Meanwhile, two markers' variation was observed in the basal-like and luminal groups (Table 5(b)). In phase II, the correlation between two (CK19 and Ki-67) markers' coexpression and pathological features (basal-like HER2^−^, ER^−^/PR^−^) was significantly different (*P* ≤ 0.001), but no correlation was found between biomarker coexpression and pathological features in phase I.

## 4. Discussion

Clinical and pathological evaluations by gene-expression profiling may provide better predictions for the development of genomic tests [20, 21]. Accordingly, until now, no markers for early diagnosis of BC have been reported on the basis of their clinical utility in BC.

RT-PCR is a powerful method to detect molecular markers in the peripheral blood of patients with BC. It has already been shown that CK19 and CEA molecular markers have good specificity [18], while CA15-3 and CEA serum markers have clinical significance and are the most widely used [22, 23]; furthermore, ER/PR and HER-2 receptors are clinically important tissue markers in BC [24]. Genetic and clinical analyses are essential for a personalized approach in the diagnosis and therapy of patients with BC [25]. Furthermore, the most important predictor of a good therapeutic regimen could be the molecular subtype that it is targeted towards [26]. Therefore, in this study, we evaluated CK19, hMAM, CEA, MUC, Myc, Ki-67, ErbB2, and ER biomarker levels in patients with BC. While the function of these markers has individually been recognized and their role in pathogenesis is known, we here assessed marker expression and variation through different stages of BC and after therapy. The histopathological status of ER, PR, and HER2, which has been recognized, can better define the clinical value of molecular classification. In this evaluation, pathological features of the different stages were also considered.

We found that CK19, Ki-67, Myc, and CEA were significantly expressed in the patients. Variation in biomarker expression was observed, especially when considering the coexpression of markers; variation in the coexpression of three markers (CK19, Ki-67, and Myc) was demonstrated in stage I and after treatment. Furthermore, coexpression of CK19 and Ki-67 drastically decreased after treatment. Marker variation was also observed on the basis of histopathological classification based on ER/PR/HER status; in the histopathologically classified basal-like group, downregulation of two markers (CK19 and Ki-67) was also observed. Early detection of cancer in patients leads to better recovery rates and survival than late detection of advanced cancer in patients [27]; using the aforementioned strategy, disease-free and overall survival could be evaluated.

Molecular technology can be used to characterize the molecular subtypes of BC. This was a pilot study for the classification of patients with BC based on the molecular approach, and molecular markers in patients with BC and modulation in their expression were determined for the first time. Further studies are needed to analyze the detailed molecular differences between BC subtypes [11], which could reveal new therapeutic targets [9]. The effect of chemotherapy in different molecular type of tumors could be considered regarding marker variation, which will lead to new therapeutic approaches to obtain the best BC treatment outcomes. In addition, molecular marker coexpression may yield a specific signature that could be used for the development personalized therapy.

## Figures and Tables

**Table 1:** Clinical and pathological characteristics of the patients.

Characteristics	*N*	%
Overall series of patients	30	100
Mean age = 45 (range: 31–56 years)		
Age < 45	16	53.33
Age ≥ 45	14	46.66
Menopausal status		
Pre	10	33.33
Post	20	66.66
Histological diagnosis		
Invasive ductal carcinoma	26	86.66
Invasive lobular carcinoma	2	6.66
Other subtypes^*∗*^	2	6.66
Tumor size		
≤2 cm	12	40
>2 cm	18	60
Stage		
I	11	36.33
II and III	19	63.66
Lymph node involvement		
Negative	20	66.66
Positive	10	33.33
Nuclear grade		
I	4	13.33
II	16	53.33
III	10	33.33
Receptor status		
ER^+^	16	53.33
ER^−^	14	46.66
Immunohistochemical profile		
Luminal A/B Basal-like	16 14	53.33 46.66
Mean follow-up = 3 years		

*N*: number of subjects; *∗* includes mucinous and papillary carcinomas; ER: estrogen receptor; luminal A: HER^+^, ER^+^/PR^+^; luminal B: HER2^−^/ER^+^/PR^+^; basal-like: HER2^−^, ER^−^/PR^−^.

**Table 2:** Sequences of biomarker primers used in this study (F, forward primer; R, reverse primer).

Gene	Marker description	Ref.
CK19	*General marker upregulated in epithelial tumor* F 5′-ATGAAAGCTGCCTTGGAAGA-3′ R 5′-TGATTCTGCCGCTCACTATCAG-3′	[9]
hMAM	*Secretory epithelial protein overexpressed in breast cancer * F 5′-CCATGAAGTTGCTGATGGTC-3′ R 5′-TCAGAGTTTCATCCGTTTGG-3′	[10]
HER2/neu	*Receptor tyrosine kinase associated with signal transduction* F 5′-GGATATCCAGGAGGTGCAGGGTAC-3′ R 5′-CCTGTGAGGCTTCGAAGCTGCAGCT-3′	[11]
MUC1	*Membrane mucin present in epithelia, participating in cellular signaling* F 5′-CGTCGTGGACATTGATGGTACC-3′ R 5′-GGTACCTCCTCTCACCTCCTCCAA-3′	[12]
Myc	*Transcription factor involved in apoptosis and cell proliferation* F 5′-CAGCTGCTTAGACGCTGGATTT-3′ R 5′-ACCGAGTCGTAGTCGAGGTCAT-3′	[13]
Ki-67	*Marker for proliferating cells* F 5′-ATCGTCCCAGGTGGAAGAGTT-3′ R 5′-ATAGTAACCAGGCGTCTCGTGG-3′	[14]
ErbB2	*Oncogene code for epidermal growth factor receptor (GFR)* F 5′-CTGGTGACACAGCTTATGCCCT-3′ R 5′-ATCCCCTTGGCAATCTGCA-3′	[12]
ER*α*/*β*	*Estrogen receptor alpha and beta* F 5′-TGCTTCAGGCTACCATTATGGAGTCTG-3′ R 5′-GTCAGGGACAAGGCCAGGCTG-3′ F 5′-TTTAAAGAAGCATTCAAGGACATAATG-3′ R 5′-GAAGTGTGGCTCCCGGAGAGAGAG-3′	[14]
CEA	*Glycoprotein involved in cell adhesion* F 5′-TCTGGAACTTCTCCTGGTCTCTCAGCTGG-3′ R 5′-TGTAGCTGTTGCAAATGCTTTAAGGAAGAAGC-3′ F 5′-GGGCCACTGTCGGCATCATGATTGG-3′ R 5′-TGTAGCTGTTGCAAATGCTTTAAGGAAGAAGC-3′	[15]
GAPDH	*Housekeeping gene* used to normalize the expression F 5′-GGTCGGAGTCAACGGATTTG-3′ R 5′-ATGAGCCCCAGCCTTCTCCAT-3′	[9]

**Table:** (a) Biomarker expression in healthy women and patients in phases I (prior to surgery) and II (after treatment) was compared and *P* value was calculated

	Healthy women		Patients (phase 1)		Patients (phase II)		*P* value	
Biomarker expression	*N*	%	*N*	%	*N*	%	*Phase I compared to healthy women*	*Phase I compared to phase II*
CK19	22	73	28	93	17	56.6	**0.012**	**≤ 0.001**
hMAM	18	60	17	56	17	56.6	NS	**—**
HER2	0	0	0	0	0	0	**—**	**—**
MUC1	28	93	30	100	30	100	NS	**—**
Myc	10	33	15	50	4	13.3	**0.023**	**≤ 0.001**
Ki-67	19	63	25	83	13	43.3	**0.012**	**≤ 0.001**
ErbB2	12	40	15	50	15	50	NS	**—**
ER	19	63	20	67	17	58.3	NS	*NS*
CEA	11	36	15	50	14	46.6	*0.043*	NS

*N*: number of subjects; *P* value ≤ 0.05 was considered statistically significant; NS: not significant.

**Table:** (b) Biomarker expression in phases I (prior to surgery) and II (after treatment) and its correlation with the stage of disease was compared in patients and *P* value was calculated

Biomarker expression	Phase I					Phase II				
	Stage I		Stages II/III		*P* value	Stage I		Stages II/III		*P* value
	*N*	%	*N*	%		*N*	%	*N*	%	
CK19	11	39	17	61	NS	5	29	12	71	NS
hMAM	10	59	7	41	**0.004**	9	53	8	47	**0.034**
HER2	0	0	0	0	**—**	0	0	0	0	**—**
MUC1	11	37	19	63	**—**	11	37	19	63	**—**
Myc	10	67	5	33	**0.001**	2	50	2	50	**—**
Ki-67	11	44	14	56	NS	8	60	5	40	**0.013**
ErbB2	10	67	5	33	**0.001**	0	0	15	100	**≤0.001**
ER	10	50	10	50	**—**	10	57	7	42.9	**0.004**
CEA	8	53	7	47	0.058	8	57	6	42.9	**0.029**

*N*: number of positive subjects; %: positivity percentage; *P* value ≤ 0.05 was considered statistically significant; NS: not significant.

**Table:** (a) Biomarker coexpression in blood from healthy women and patients in phases I (before treatment) and II (after treatment)

Biomarker coexpression	Healthy women		Patients				*P* value		
			Phase I		Phase II				
	(*N* = 30)		(*N* = 30)		(*N* = 30)				
	Positive		Positive		Positive		*P*1	*P*2	*P*3
CK19, Ki-67, Myc, CEA	2	6**%**	13	43**%**	0	0**%**	**0.00**	NS	**0.00**
CK19, Ki-67, Myc	2	6**%**	13	43**%**	0	0**%**	**0.00**	NS	**0.00**
CK19, Ki-67	19	63**%**	22	73**%**	10	33**%**	NS	**0.02**	**0.00**

*P* value ≤ 0.05 was considered statistically significant;*P*1 was considered between phase I and healthy women; *P*2 was considered between phase II and healthy women; *P*3 was considered between phases I and II.

**Table:** (b) Biomarker coexpression and its correlation with stage of disease in phases I (before surgery) and II (after treatment)

Biomarker coexpression	Phase I					Phase II				
	Stage I		Stages II/III		*P* value	Stage I		Stages II/III		*P* value
	*N*	%	*N*	%		*N*	%	*N*	%	
CK19, Ki-67, Myc, CEA	10	77	3	23	**≤0.001**	0	0	0	0	**—**
CK19, Ki-67, Myc	10	77	3	23	**≤0.001**	0	0	0	0	**—**
CK19, Ki-67	11	50	11	50	**0.006**	5	50	5	50	NS

*P* value ≤ 0.05 was considered statistically significant.

**Table:** (a) Marker expression in phase I (before surgery)

Biomarkers expression	Luminal HER2^−/+^, ER^+^/PR^+^		Basal-like HER2^−^, ER^−^/PR^−^		*P* value
	*N*	%	*N*	%	
CK19	14	50	14	50	0.17
Myc	10	66.7	5	33.3	0.14
Ki-67	15	60	10	40	0.10
CEA	10	66.7	5	33.3	0.14

*P* value ≤ 0.05 was considered statistically significant.

**Table:** (b) Marker coexpression in phases I (before surgery) and II (after treatment)

Biomarker coexpression	Phase I				*P* value	Phase II				*P* value
	Luminal like HER2^−/+^, ER^+^/PR^+^		Basal-like HER2^−^, ER^−^/PR^−^			Luminal like HER2^−/+^, ER^+^/PR^+^		Basal like HER2^−^, ER^−^/PR^−^		
	*N*	%	*N*	%		*N*	%	*N*	%	
CK19, Ki-67, Myc, CEA	8	61.5	5	38.5	0.4	0	0	0	0	—
CK19, Ki-67, Myc	8	61.5	5	38.5	0.4	0	0	0	0	—
CK19, Ki-67	12	54.5	10	45.5	0.8	10	100	0	0	**≤0.001**

*P* value ≤ 0.05: correlation was considered statistically significant.
